# Implementation of Digital Seamless Nutrition Care throughout the Treatment Course for Patients with Head and Neck Cancer: A Process Evaluation

**DOI:** 10.1016/j.cdnut.2026.107677

**Published:** 2026-03-21

**Authors:** Frida Severinsen, Cecilie Varsi, Lene Frost Andersen, Christine Henriksen, Mari Mohn Paulsen

**Affiliations:** 1 Department of Nutrition, Institute of Basic Medical Sciences, University of Oslo, Oslo, Norway; 2 Faculty of Health and Social Sciences, University of South-Eastern Norway, Borre, Norway; 3 Department of Food Safety, Norwegian Institute of Public Health, Oslo, Norway; 4 Centre for Sustainable Diets, Norwegian Institute of Public Health, Oslo, Norway

**Keywords:** digital health, cancer, eHealth, implementation science, malnutrition

## Abstract

**Background:**

Nutritional interventions may reduce the prevalence of malnutrition in patients with head and neck cancer; however, implementation often fails due to barriers at various levels. Process evaluations can provide an understanding of implementation outcomes, mechanisms of impact, and context and provide information about feasibility and acceptability.

**Objectives:**

To perform a process evaluation of the Nutrition Throughout the Treatment Course (NUTREAT) intervention, including use of the MyFood app and web report. Specifically, we aimed to evaluate *1*) the acceptability, adoption, appropriateness, feasibility, and fidelity of the intervention, *2*) the implementation plan, and *3*) patient adherence to the intervention.

**Methods:**

A mixed methods approach was applied, including individual interviews, a focus group discussion, log data from electronic patient records (EPRs), an experience questionnaire, and information from the MyFood web report. Proctor’s implementation outcomes guided the development of the interview guide and analysis. Themes were deductively established using codebook thematic analysis. The implementation plan was evaluated through logs of completed activities. Log data from EPRs were collected to evaluate fidelity among healthcare professionals (HCPs), and information from the MyFood web report informed patient adherence to the intervention.

**Results:**

Individual interviews were conducted with patients (*n* = 8), registered nurses (*n* = 3), and leaders (*n* = 2), and a focus group discussion was conducted with registered dietitians (*n* = 6). Patients, registered nurses, registered dietitians, and leaders found the intervention acceptable, appropriate, and feasible. Patient adherence was high, whereas fidelity among HCPs was low.

**Conclusions:**

The NUTREAT intervention increased patients’ awareness of their own nutritional requirements and was considered helpful by HCPs in preparing for patient consultations. However, fidelity among HCPs was low. Future efforts should focus on identifying strategies to improve fidelity among HCPs and study the long-term sustainability of the intervention.

This study was registered at clinicaltrials.gov as NCT05997329.

## Introduction

Head and neck cancer (HNC) is a collective term for cancers in the oral cavity, sinonasal cavity, pharynx, and larynx [1]. Surgery, following adjuvant radiotherapy or concurrent chemoradiotherapy is the most common treatment for HNC [2]. Patients with HNC have a high prevalence of malnutrition [3], often attributed to the presence of nutrition impact symptoms prior to [4], during [5], and after treatment [6], indicating a need for closer nutritional monitoring and follow-up for patients with HNC throughout the treatment course.

Several nutritional interventions in patients with HNC have shown to reduce the prevalence of malnutrition and severity of weight loss [7,8] in addition to improving treatment tolerance [8]. Furthermore, various digital health interventions have been developed, particularly focusing on symptom tracking and selfcare, which have demonstrated that active digital treatment support positively impacts physical symptoms in patients with HNC [9].

Despite the increasing development of both digital health and nutritional interventions, their implementation into clinical practice often fails due to barriers at various levels, including the patient, provider or group, and organizational level [10]. Process evaluations seek to provide a deeper understanding of implementation, mechanisms of impact (i.e., how an intervention produces its intended outcomes), and contextual factors to determine whether an intervention has been successfully implemented in a given setting [11,12]. Moreover, process evaluations can provide information about the feasibility and acceptability of an intervention, as well as assisting in the interpretation of the effect outcomes [13].

As a response to the need for better tools to follow-up patients at risk of malnutrition, the MyFood system was developed, consisting of an app to assess and monitor nutritional intake among hospitalized patients and a web report to provide decision support to healthcare professionals (HCPs) [14]. The MyFood system has previously been evaluated in both hospital [14] and home settings [15]. In both settings, dietary recordings in the MyFood app showed high agreement with validated reference methods, and use of the MyFood system was associated with a reduced risk of malnutrition among hospitalized patients [16]. Additionally, a process evaluation was conducted to assess various implementation aspects in a hospital setting for patients with hematologic cancer, revealing that patients were more compliant with using the MyFood system than registered nurses [17].

More recently, the MyFood system was incorporated into the Nutrition Throughout the Treatment Course (NUTREAT) intervention. The NUTREAT intervention was developed to reduce the prevalence of malnutrition among patients with HNC throughout the treatment course by increasing awareness of individual dietary intake and improving the accessibility of nutrition-related information for HCPs through use of the MyFood system.

Utilizing a hybrid type 1 design, combining aspects of effectiveness and implementation as described by Landes et al. [18], the NUTREAT intervention was tested in a randomized controlled trial (RCT) (Severinsen F, Henriksen C, Varsi C, Gjelstad IMF, Jager-Wittenaar H, Andersen LF, Paulsen MM). Prior to the RCT, a pre-implementation study was conducted [19], in which barriers and opportunities associated with the implementation of the NUTREAT intervention were identified to inform the development of an accompanying plan for the subsequent implementation of the NUTREAT intervention. The implementation plan included a range of 16 carefully selected implementation strategies [20], defined as “methods or techniques used to enhance the adoption, implementation, and sustainability of a clinical program or practice” [21]. These strategies were adapted from the Expert Recommendations for Implementing Change (ERIC) project, which includes a compilation of 73 discrete implementation strategies [20].

In the present study, we conducted a process evaluation of the NUTREAT intervention alongside an RCT. Specifically, we aimed to evaluate *1*) the acceptability, adoption, appropriateness, feasibility, and fidelity of the intervention, *2*) the implementation plan, and *3*) patient adherence to the intervention.

## Methods

### Study design

To evaluate implementation-related aspects of the NUTREAT intervention, a process evaluation was conducted using a mixed methods approach. We followed the Consolidated Criteria for Reporting Qualitative Research (COREQ) guidelines [22] when reporting qualitative findings, in addition to following the Consensus-based Process Evaluation Reporting Guideline for Public Health Intervention Studies (CONPHES) [23] when reporting the process evaluation.

### Study setting and treatment course for patients with HNC

The study was conducted at a department for Head and Neck Oncology at a large university hospital in Norway. At this hospital, the treatment course for patients with HNC is primarily outpatient-based, lasting approximately 18 wk, including 6 wk of radiotherapy and 6- and 12-wk follow-ups after completed radiotherapy. An important aspect of the treatment course for patients with HNC is the established follow-up by a registered dietitian (RD) at specific time points throughout the treatment course. However, this applies only to the outpatients, whereas inpatients are referred to an RD when clinically indicated.

### The NUTREAT intervention

The NUTREAT intervention involved the use of the MyFood app for dietary assessment and monitoring [14] throughout the treatment course for patients with HNC and the follow-up of these dietary assessments during consultations at the hospital, with additional referral to an RD in cases of insufficient dietary intake.

**FIGURE 1:**
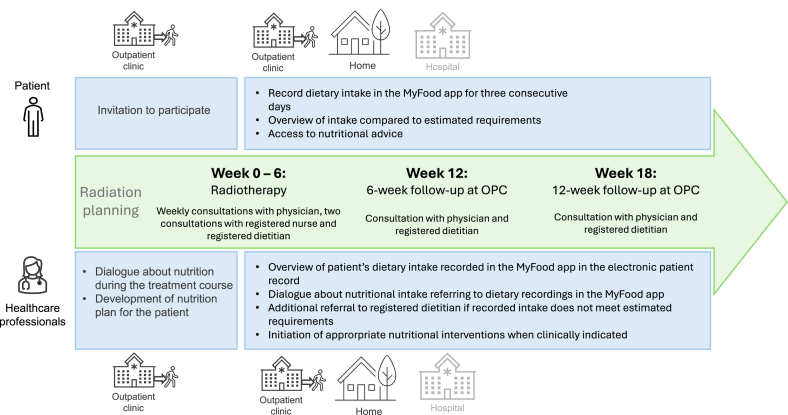
Overview of the NUTREAT intervention and the treatment course for patients with head and neck cancer in the outpatient clinic, home, and hospital. The hospital setting is shaded in light grey as not all patients are admitted to the hospital throughout their treatment course. OPC, outpatient clinic.

The NUTREAT intervention required patients to record their dietary intake in the MyFood app throughout their treatment course for 3 consecutive days over a total of 4 recording periods, in total 12 recording days, regardless of whether they were followed as inpatients or outpatients. The recording periods were conducted prior to consultations with physicians, RDs, or registered nurses at the outpatient clinic, and a key component of the intervention was to increase awareness of nutritional requirements among the patients. An overview of the treatment course and the NUTREAT intervention is presented in Figure 1.

**FIGURE 2:**
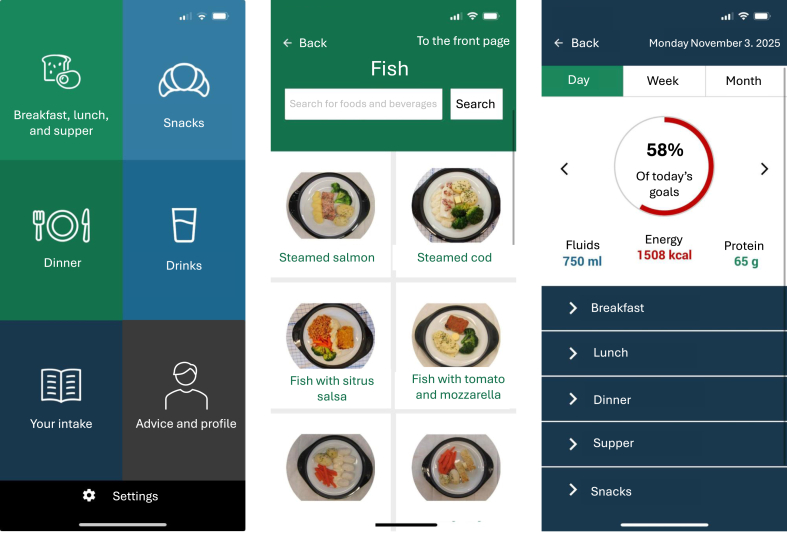
Screenshots of the MyFood app. From the left: *1*) main menu of the dietary recording function; *2*) menu for recording the dinner meal; and *3*) evaluation of recorded intake compared to the estimated requirements for energy, protein, and fluid.

The MyFood app for smartphone or tablet devices includes recording of dietary intake, body weight, and nutrition-related symptoms [14]. Upon registration in the MyFood app, patients enter their weight, height, and age, which results in an automatic calculation of their estimated requirements for energy, protein, and fluids. These calculations are based on an energy requirement of 30 kcal/kg/d, with adjustments made for patients with a BMI >25 kg/m^2^ for their requirements to correspond to those with a BMI of 25 kg/m^2^ [24,25]. For protein and fluids, the requirements are based on 1.2 g/kg body weight/d and 30 mL/kg body weight/d, respectively [24,25]. The recorded intake in the MyFood app is continuously compared to the patients’ estimated requirements, providing an overview of the degree to which the requirements are fulfilled each day, as presented in Figure 2.

Daily summaries of nutrition requirement fulfillment are automatically transferred to the MyFood web report, which HCPs access using their personal identification number [14,16]. Previous studies have identified lack of electronic patient record (EPR) integration and repeated log-ins to the web report as barriers to routine use among HCPs [16,17]. Thus, for the NUTREAT intervention, a project worker accessed the web report and pasted the information into designated dietary recording notes in the EPR for each patient after each dietary recording period to simulate a potential solution for future integration of the MyFood system in the EPR. The dietary recording notes from the dietary recording periods differ from the regular clinical notes written by RDs, physicians, and registered nurses, as they provide a clear overview of the total energy, protein, and fluid intake for each day as well as a mean across the 3-d recording periods. In addition, they present the patient’s intake as a percentage of estimated requirements for each day and each recording period. In contrast, regular clinical notes are typically more comprehensive and include a structured overview of assessment, diagnosis, and nutritional intervention, as well as a plan for monitoring and evaluation.

In the NUTREAT study, physicians and registered nurses working with patients included in the study were instructed to review the dietary recording notes prior to their consultations with the patient to acquire information about the patient’s dietary intake and to have a dialogue with patients regarding the recorded intake in the MyFood app. If the recorded intake was <75% of the estimated energy requirements for the 3 recording days, physicians and registered nurses were instructed to provide an additional referral to an RD. The RDs were informed that while they could use the dietary recording notes in their patient consultations, they were not required to do so as they already have a standardized nutritional follow-up of the patients, including a dietary assessment.

### Implementation activities as outlined in the implementation plan

Based on the results from the pre-implementation study [19], which was conducted prior to the present study, an implementation plan including selected strategies from the ERIC project [20] was developed to guide the implementation of the NUTREAT intervention into the treatment course for HNC. The implementation plan was used as a foundation for all activities performed to enhance the uptake of the NUTREAT intervention among patients and HCPs.

The first part of the implementation plan involved assessing the current situation, identifying barriers and opportunities, and tailoring implementation strategies. The second part included strategies aiming to prepare the hospital setting to implement the NUTREAT intervention, including informational meetings with the involved HCPs and leaders as well as developing and distributing informational materials to HCPs about recruitment, the intervention, and how to perform follow-up procedures. An implementation advisor facilitated this process by providing expert guidance to align these strategies with the specific context within the NUTREAT intervention. The third part of the implementation plan involved strategies for implementing the NUTREAT intervention during the RCT. This included regular e-mail correspondence and physical meetings with leaders and HCPs. Adherence to the implementation plan was assessed using a hardcopy logbook for documenting completed activities. The application of the various implementation strategies in the implementation plan is described in Table 1. For more details on the implementation plan, see Supplementary File 1.

**TABLE 1:** Implementation strategies adapted from the ERIC project [20] and their application in the implementation plan for the NUTREAT intervention.

Description	Implementation strategies	Application of implementation strategies in the present study
Part 1		
Assessment of the current situation and planning the implementation effort	Use evaluative and iterative strategies • Assess barriers and opportunities • Review previous literature Adapt and tailor to context • Tailor strategies	• Pre-implementation study including individual interviews • Reviewing identified barriers, opportunities, and implementation strategies identified in previous studies of the MyFood system • Tailoring strategies based on results from the pre-implementation study [19] and the review of previous studies using MyFood • Preparing the implementation process by developing an implementation plan
Part 2		
Preparations at the hospital and conducting educational meetings with healthcare professionals	Train and educate stakeholders • Conduct educational meetings • Develop educational materials • Distribute educational materials Develop stakeholder interrelationships • Identify and prepare champions • Recruit, designate, and train for leadership • Use an implementation advisor	• Conducting educational meetings with registered nurses, physicians, and registered dietitians • Developing and distributing informational pamphlets about the study to registered nurses, physicians, and registered dietitians • Developing and distributing a user manual for the MyFood system • Appointing registered nurses at the outpatient clinic as “champions” • Involving hospital leaders in planning the randomized controlled trial • Appointing the second author (CV) as an implementation advisor
Part 3		
Conducting the randomized controlled trial, i.e., implementing the NUTREAT intervention	• Audit and provide feedback • Organize clinician team meetings Support clinicians • Facilitate relay of clinical data to providers • Remind clinicians • Conduct ongoing training Provide interactive assistance • Provide clinical supervision • Provide local technical assistance	• Conducting formal and informal meetings with involved healthcare professionals regarding progression and preliminary results in the randomized controlled trial • Distributing newsletters regularly by e-mail to involved healthcare professionals • Ensuring availability of project workers for support through a project phone • Conducting regular correspondence with leaders and involved healthcare professionals to remind clinicians of the intervention

Abbreviations: ERIC, Expert Recommendations for Implementing Change; NUTREAT, Nutrition Throughout the Treatment Course.

### Study population

A total of 125 patients with HNC were enrolled in the RCT and randomly assigned to receive the NUTREAT intervention, including use of the MyFood app (*n* = 62), or standard care (*n* = 63). For the process evaluation in the present study, the first 11 patients who had completed the NUTREAT intervention in the RCT were invited to participate in individual interviews through purposive sampling [26], of whom 8 agreed to participate. Of the 8 participants in the process evaluation, 50% were male and their mean age was 64 y, ranging from 55 to 74 y. HCPs in the process evaluation included 6 RDs, all of whom were women; 3 registered nurses, of whom two-thirds were women; and 2 leaders, who were both women. The HCPs were also purposively sampled [26] based on their work at the departments where the RCT was conducted, with RDs and registered nurses being recruited through their respective leaders, whereas the leaders were directly approached with an invitation to participate.

The adequacy of the sample size was determined using the “information power” principle [27], which is considered a more appropriate method for determining sample size in qualitative studies than traditional saturation, as it recognizes that fewer participants can yield valuable and relevant insights if their contributions are rich and aligned with the study objectives, provided that the study objectives are clearly stated [27]. Given the small number of HCPs working in the relevant departments, this approach was adopted, and multiple professional groups were included to ensure a rich data foundation and enhance credibility.

### Outcome measures

Patient *adherence* to the intervention was defined as the number of completed recording days within the 4 predetermined recording periods, divided by the total of 12 prescribed recording days. Furthermore, an assessment of the total number of recording days completed throughout the treatment course was conducted by counting the total number of recoding days completed from the web report.

Implementation outcomes described by Proctor et al. [28] were used to evaluate the implementation of the NUTREAT intervention and adherence to the implementation plan. The implementation outcomes included *acceptability*, *adoption*, *appropriateness*, *feasibility*, and *fidelity* [28]. These implementation outcomes are proposed to have the potential to capture contextual factors important for evaluating implementation effect among providers, improve comprehension of the implementation process, and enable assessment of implementation strategy effect [28]. A detailed description of each of the implementation outcomes addressed in this process evaluation is presented in Table 2.

**TABLE 2:** Overview of the applied implementation outcomes described by Proctor et al. [28] and how they were addressed in the process evaluation.

Implementation outcome	Description	Data collection
Acceptability	Stakeholders’ perceptions of the degree to which an intervention is acceptable, tolerable or satisfactory [28]	• Individual interviews with patients, leaders, and registered nurses • Focus group discussion with registered dietitians • Patient experience questionnaire
Adoption	The initial intention or decision to try or use an intervention. Can also be referred to as “uptake” [28]	• Individual interviews with leaders and registered nurses • Focus group discussion with registered dietitians
Appropriateness	The degree to which an intervention is considered relevant, appropriate, or compatible with the providers, recipients, or setting to which it is to be implemented [28]	• Individual interviews with patients, leaders, and registered nurses • Focus group discussion with registered dietitians • Patient experience questionnaire
Feasibility	The degree to which an intervention can be successfully implemented or used within the setting [28]	• Individual interviews with patients, leaders, and registered nurses • Focus group discussion with registered dietitians
Fidelity	How closely an intervention is implemented or provided in line with the original protocol [28]; in this study, referring directly to the delivery of the intervention from healthcare professionals	• Individual interviews with leaders and registered nurses • Focus group discussion with registered dietitians • Log data from the electronic patient record

### Data collection

Data sources in this process evaluation included both qualitative (individual interviews and focus group discussion) and quantitative information (questionnaire, log data from the EPR, and information from the MyFood web report).

#### Individual interviews

Semistructured individual interviews were conducted with patients, registered nurses, and leaders using 3 separate interview guides (Supplementary File 2) to explore their experiences regarding the implementation of the NUTREAT intervention. The interview guides were developed by the authors based on the implementation outcomes described by Proctor et al. [21], as presented in Table 2.

The interview guide for the patients was pilot tested in 2 interviews; however, no changes were made to the interview guide, and the interviews were included in the final data material. The interviews with patients and registered nurses were conducted by a project worker who had received training in conducting interviews. No prior relationship existed between the researcher and the participants. Interviews with the leaders were conducted by the first author (FS), who was already well acquainted with the leaders and had experience in conducting interviews. The interviewers were all researchers and held a master’s or a PhD degree in nutrition. All participants were informed about the researchers’ roles and study aims before providing consent.

The interviews with patients were conducted by telephone after the patients’ 12-wk follow-up, once they had completed the intervention. Interviews with the leaders and registered nurses were conducted in-person in a meeting room at the hospital toward the end of the recruitment period of the RCT. The interviews were conducted between August 2024 and March 2025 and lasted between 24 and 71 min, with a mean of 44 min.

The individual interviews were audiotaped and transcribed verbatim using *Autotext*, an automatic transcription tool of speech to text developed by the University of Oslo.

#### Focus group discussion

The focus group discussion was conducted with the RDs by the same project worker who conducted the interviews with registered nurses and patients, to inform the implementation outcomes as presented in Table 2. The last author (MMP) assisted in the focus group by taking notes. The focus group followed a semistructured interview guide (Supplementary File 3) and was conducted in a meeting room at the hospital in October 2024, lasting for 1 h and 18 min. The focus group discussion was audiotaped and transcribed verbatim, as described for the individual interviews above.

#### The MyFood web report

Information regarding the patients’ use of the MyFood app was collected from the web report of the MyFood system [16], which provided an overview of the recorded dietary intake in addition to the number of completed recording days and periods.

#### Electronic patient record

Data that HCPs accessed in the dietary recording notes in the EPR were gathered using a logging feature in the EPR system, which provided an overview of who had accessed each note. The number of unique HCPs who had accessed the notes were counted, in addition to counting the number of dietary recording notes each individual HCP accessed. Furthermore, a search function in the EPR was used to find notes from HCPs that had referred to the dietary recording notes. This information was used to inform the implementation outcome, *fidelity*.

#### Experience questionnaire

An experience questionnaire applied in previous studies of the MyFood system [15,16] was used to explore the outcome measures *acceptability* and *appropriateness.* The questionnaire consisted of 5 claims: *1) I found the MyFood app easy to use; 2) I found the food and beverages I wanted to record; 3) I managed to record the correct amount; 4) I needed to obtain new knowledge to use the MyFood app;* and *5) I became more aware of my own nutritional requirements through using the MyFood app*. The questions were ranged on a 5-point Likert scale, in addition to an open-text field for any additional comments. Patients in the intervention group completed the questionnaire at the end of the RCT.

### Data analysis

Transcripts from the individual interviews and focus group discussion were analyzed using codebook thematic analysis [29]. The software NVivo 15 (QSR International) was used for sorting the themes. The transcripts were initially read by the first author (FS) for familiarization with the data. Next, themes were deductively established to cover the outcomes *acceptability, adoption, appropriateness, feasibility,* and *fidelity.* Finally, the themes were re-evaluated and resorted if deemed necessary. The first (FS), second (CV), and last (MMP) authors were involved in all parts of the data analysis. The log data from the EPR and the information from the MyFood web report was analyzed in Microsoft Excel 365 using descriptive statistics.

### Ethical considerations

The study was conducted in accordance with the Declaration of Helsinki and was approved by the data protection authority at the hospital and the Regional Committees for Medical and Health Research Ethics (2023/569956). The study was registered in the National Institutes of Health Clinical Trials database (reference identification: NCT05997329) and the Norwegian Agency for Shared Services in Education and Research (reference identification: 923757). Written informed consent was obtained from all participants, and the interview recordings were securely stored in “Services for sensitive data” hosted by the University of Oslo, to which only dedicated project members have access. Interview transcripts were deidentified with unique participation identification numbers.

## Results

The results draw upon data from individual interviews with 8 patients, 2 leaders, and 3 registered nurses, a focus group discussion with 6 RDs, the MyFood web report, dietary recordings from the EPR, and an experience questionnaire. The results are discussed in relation to Proctor’s implementation outcomes [28].

### Patient adherence to the NUTREAT intervention

On mean, adherence to the NUTREAT intervention was 82% (minimum 0%, maximum 100%). An overview of the proportion of patients completing each of the recording days within each of the 4 recording periods in the MyFood app is presented in Table 3.

**TABLE 3:** Overview of the proportion of patients completing each recording period in the MyFood app and the number of recording days completed within each period.

Recording period^1^	0 d	1 d	2 d	3 d
	*n* (%)	*n* (%)	*n* (%)	*n* (%)
1 (*n* = 61)	3 (5%)	2 (3%)	6 (10%)	50 (82%)
2 (*n* = 59)	3 (5%)	2 (3%)	4 (7%)	50 (85%)
3 (*n* = 55)	8 (15%)	1 (2%)	6 (11%)	40 (73%)
4 (*n* = 52)	12 (23%)	0 (0%)	3 (6%)	37 (71%)

1 *n* varies across the 4 recording periods due to dropouts for various reasons.

The proportion of patients completing 3 recording days was relatively stable for all 4 recording periods, although the proportion not completing any recording days increased by the third and fourth recording period. This aligns with findings from the interviews, where some patients reported decreased motivation to complete the recording periods toward the third and fourth periods due to increasing treatment side effects. Furthermore, several patients reported that they felt obligated to follow the intervention since they had agreed to participate in the study.

The total number of recording days completed ranged from 0 to 105 d. Nearly half of the patients (44%) used the MyFood app for dietary recording beyond the 4 recording periods. This was also reported during the interviews by some patients who shared that they used the MyFood app almost daily throughout and after the treatment course:

“I didn’t have to record every day, week after week and month after month all the time. It was only a few times, right? Three days, then so many times. But I made it a habit that I did it every day all week long and I continued like that.” – Patient 2

### Implementation outcomes

**FIGURE 3:**
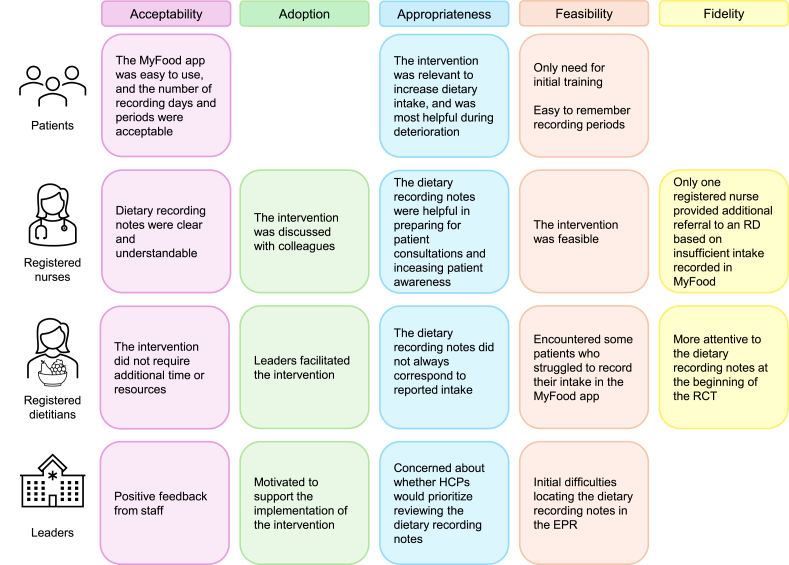
Overview of main results for each of the implementation outcomes based on Proctor et al. [28], with the rows showing the perspectives of patients, registered nurses, registered dietitians, and leaders. EPR, electronic patient record; HCP, healthcare professional; RCT, randomized controlled trial; RD, registered dietitian.

The analyses of the qualitative interview data resulted in 5 themes corresponding to 5 of the implementation outcomes described by Proctor et al. [28]. Figure 3 provides an overview of the main results for each of the outcomes, from patients, registered nurses, RDs, and leaders perspectives. These results are presented below.

#### Acceptability

Overall, patients, registered nurses, RDs and leaders reported that the NUTREAT intervention was acceptable and manageable. The patients’ perceptions of the acceptability of the MyFood app from the experience questionnaire are presented in Table 4.

**TABLE 4:** Perceived acceptability of the MyFood app among the patients (*n* = 43).

	Totally disagree *n* (%)	Disagree *n* (%)	Neutral *n* (%)	Agree *n* (%)	Totally agree *n* (%)
I found MyFood easy to use	1 (2%)	2 (5%)	5 (12%)	6 (14%)	29 (67%)
I found the food and beverages I would record	0 (0%)	5 (12%)	11 (26%)	9 (21%)	18 (42%)
I recorded the correct amount	0 (0%)	4 (9%)	9 (21%)	8 (19%)	22 (51%)
I needed to acquire new knowledge to use MyFood	28 (65%)	6 (14%)	1 (2%)	4 (9%)	4 (9%)

All the patients expressed satisfaction with the number and periods of dietary recording days in the MyFood app. Although some indicated that they could accept additional recording days, others experienced that the current number was sufficient. Furthermore, most of the patients reported that they found the MyFood app easy to use (81%), that they managed to record the correct amount of food and beverages (71%), and that they did not need to acquire new knowledge to use the app (81%). However, although a large proportion of patients reported that they found the foods and beverages they would record (61%), several indicated in the interviews and the open-text field of the questionnaire that they desired a wider selection of foods and beverages.

The leaders reported receiving positive feedback from their staff, and most registered nurses and RDs reported that they primarily received positive feedback from patients and colleagues regarding the intervention. Registered nurses and RDs expressed that the intervention did not require additional time or resources. Furthermore, the registered nurses reported finding the dietary recording notes in the EPR clear and understandable:

“No, I think it’s a bit nice that they (the NUTREAT dietary recording notes) are not as comprehensive as the registered dietitian notes. It’s a bit quicker to get an overview. They can be very good those registered dietitian notes too, because you get a lot of insight, but the patients can tell the rest themselves, in a way. If we get the quick overview of how they’re doing, they can fill in the rest.” – Registered nurse 1

#### Adoption

The registered nurses reported having informal conversations with colleagues about how they could best implement and follow-up the intervention, indicating high adoption. Furthermore, leaders reported that they considered the intervention important and that they were motivated to support its implementation. This was also highlighted in the interviews with the registered nurses and the focus group with the RDs, who experienced that their leaders were positive toward the NUTREAT intervention and focused on the execution, which also influenced their motivation, indicating high adoption among the leaders:

“But for me at least, I kind of noticed that they (the leader) were very clear that this was important for us to contribute to. And I think that for me, it may have contributed to me thinking that I have to do this. It’s possible that if they (the leader) hadn’t said anything, or if they hadn’t been positive, it would have affected our efforts.” – Registered nurse 3

#### Appropriateness

The patients considered the intervention both relevant and valuable in increasing their dietary intake throughout their treatment course. Additionally, 74% of the patients reported in the experience questionnaire that using MyFood made them more aware of their own nutritional requirements. The intervention was considered most useful during times of deterioration in eating ability, after completion of treatment, and during the recording periods. However, some patients also reported that the intervention was effective beyond the recording periods and less useful when receiving tube feeding. Two of the patients particularly liked the overview of dietary intake compared to estimated requirements in MyFood, enabling them to assess whether they reached their daily goals:

“That I had a certain number of calories that we agreed on, in a way that makes me feel that I am in control of my own intake. In a manner simple enough for a person my age who’s not well trained in apps to manage. That’s good.” – Patient 7

Registered nurses, RDs, and leaders found the intervention useful in terms of increasing the patients’ focus and awareness on their nutritional requirements and were under the impression that patients receiving the intervention had a higher dietary intake throughout the treatment course than patients who did not. Registered nurses and RDs also experienced that the intervention was useful to them in their patient follow-up as it contributed to preparing for patient consultations more efficiently and getting to the treatment part more rapidly, as it mostly provided more reliable data on the current intake and enabled them to target their advice in accordance with the challenges the patients experienced:

“Once it is recorded how much calories, proteins and fluids they have actually consumed, we can use that to make the patient consultation more efficient or targeted.” – RD 1

However, an issue with the intervention, highlighted by the RDs, was that the intake recorded in MyFood did not always correspond to what the patients reported during consultations, which in some cases made the intervention less useful. Also, when asked which HCP would be best suited to follow-up the intervention if subsequently implemented, most of the registered nurses and RDs pointed to others than themselves and their profession, and the leaders expressed concern about whether HCPs would prioritize reviewing the dietary recording notes:

“Well, it’s one more thing they (physicians, registered nurses, and RDs) must look at, so the question is whether they have time for, or choose to look at it (dietary recording notes). Prioritizing is really what it’s all about, because you have time for anything you want to have time for, or see as important.” – Leader 1

#### Feasibility

Most patients reported that they only needed initial training at baseline to be able to use the MyFood app, except for 1 patient who desired more training. Furthermore, most patients found it easy to remember recording in the MyFood app during the recording periods, although one patient reported that it was easy to forget recording their intake if not doing it immediately after consumption:

“But the first time I was a bit like that, ‘I’ll remember this later.’ Well, you don’t, it’s completely wrong. So, I had to learn to do it almost as soon as I’d had a cup of coffee or a glass of water, otherwise it would slip out.” – Patient 3

The leaders experienced that the intervention was feasible for their employees. However, one of the leaders reported receiving feedback that HCPs initially found it difficult to locate the dietary recording notes in the EPR in the myriads of other notes. Based on this feedback, changes were made to the naming of the dietary recording notes to make them easier to find. Registered nurses and RDs also experienced that the intervention was feasible both for themselves and for most patients, although some of the RDs had encountered patients who had struggled to complete the recording periods:

“I think I’ve spoken to 1 or 2 who hadn’t received a complete registration because they didn’t manage to do it, or they didn’t find what they were supposed to record.” – RD1

#### Fidelity

An important aspect of the NUTREAT intervention was that registered nurses and physicians were instructed to review the dietary recording notes prior to consultations and provide patients with an additional referral to an RD in cases of low dietary intake recorded in the MyFood app. As presented in Table 5, a substantial proportion of patients did not fulfill ≥75% of their requirements for energy, yet only 1 registered nurse reported contacting an RD regarding a patient with insufficient intake, indicating low fidelity with this part of the intervention.

**TABLE 5:** Overview of the proportion of patients meeting ≥75% of their requirements for energy, protein, and fluids, number of dietary record notes (*n* = 199) for each recording period, and number of healthcare professionals accessing the dietary record notes.

	Period 1	Period 2	Period 3	Period 4
Proportion of patients meeting ≥75% of requirements				
Energy, *n* (%)	37 (64%)	31 (55%)	31 (66%)	34 (83%)
Protein, *n* (%)	35 (60%)	21 (38%)	29 (62%)	32 (76%)
Fluids, *n* (%)	29 (50%)	30 (54%)	20 (43%)	29 (69%)
Total number of dietary record notes from NUTREAT uploaded to the EPR	55	55	47	42
Number of dietary record notes entered once or more, *n* (%)	54 (98%)	51 (93%)	45 (96%)	35 (83%)
Number of dietary record notes not entered, *n* (%)	1 (2%)	4 (7%)	2 (4%)	7 (17%)
Number of unique HCPs accessing dietary record notes	82	86	71	36
HCPs accessing 1 dietary record note, *n* (%)	53 (65%)	60 (70%)	48 (68%)	25 (69%)
HCPs accessing 2–4 dietary record notes, *n* (%)	23 (28%)	22 (26%)	19 (27%)	9 (25%)
HCPs accessing 5–9 dietary record notes, *n* (%)	5 (6%)	4 (5%)	4 (6%)	2 (6%)
HCPs accessing ≥10 dietary record notes, *n* (%)	1 (1%)	0 (0)	0 (0)	0 (0)

Abbreviations: EPR, electronic patient record; HCP, healthcare professional; NUTREAT, Nutrition Throughout the Treatment Course.

A total of 199 dietary recording notes were uploaded and available to HCPs in the EPR. Findings from the log data regarding who had accessed the dietary recording notes in the EPR showed that most of the HCPs accessed only 1 to 4 dietary recording notes, whereas fewer HCPs accessed >5 notes. These findings were supported in the interviews, with registered nurses and RDs reporting that they used the dietary recording notes sporadically or when they found it useful for their consultations. An overview of the number of unique HCPs accessing the dietary recording notes each recording day within each period is presented in Table 5.

Dietary recordings from the MyFood app in the EPR were referred to in a total of 67 clinical notes, of which 62 were from RDs, 3 from registered nurses, and 2 from physicians, indicating a varying degree of fidelity with this part of the intervention among HCPs. The RDs were informed that they could use the dietary record notes if they wished, and both log data and interview data indicated high fidelity among the RDs. However, the RDs expressed that they were more attentive to the dietary recording notes at the beginning of the RCT, while they eventually stopped noticing them in the EPR:

“Eventually, you kind of don’t see it in the record anymore (the dietary recording notes). You don’t notice it.” – RD 3

In total, approximately 60 unique HCPs were involved in the care of patients with HNC during the study. The high number of staff accessing the dietary recording notes reflects the frequent staff turnover within the patient group. Furthermore, for 2 of the patients completing only 1 recording day, notes were not created. Thus, there were small deviations in terms of the total number of patients completing 1 to 3 recording days and the total number of dietary recording notes available.

## Discussion

This study reported on a process evaluation of the NUTREAT intervention throughout the treatment course for patients with HNC, conducted concurrently with an RCT. The intervention included digital nutrition monitoring. This comprised recording of patients’ dietary intake in the MyFood app, comparison of intake with estimated requirements, providing information to HCPs regarding the patient’s nutritional status through dietary recording notes in the EPR based on the MyFood web report, and additional referral to an RD for necessary nutritional interventions for patients with insufficient dietary intake recorded in the MyFood app.

Overall, patients, registered nurses, RDs, and leaders reported that the NUTREAT intervention was acceptable. All participants found the intervention useful in terms of increasing patients’ awareness of their nutritional requirements, and the adoption was high among the leaders, registered nurses, and RDs. The intervention was considered feasible by most of the participants, and adherence to the intervention was relatively high among the patients, whereas the fidelity was mostly low among HCP staff. Nevertheless, leaders, registered nurses, and RDs were positive toward implementing the intervention as a permanent part of the treatment course.

### Patient adherence to the NUTREAT intervention

Patient adherence to the NUTREAT intervention was relatively high; however, as emerged from both the interviews and the MyFood web report, it decreased among some patients over time. Possible explanations for this may be that the patients no longer felt the need to record their intake due to improvements in dietary intake or that the time interval between the third and fourth recording periods was too long for them to perceive value in the recordings. This aligns with findings from a study investigating user engagement with technology, which observed that poor engagement could occur from reduced interaction over time [30]. Nevertheless, almost 50% of the patients used MyFood for dietary recording beyond the 4 predetermined recording periods, indicating that they probably stayed motivated and experienced value in using it throughout their treatment course. In a previous process evaluation of the MyFood system in a hospital setting, the adherence among the patients was also high, with patients reporting that they found it easier to reach their nutritional requirements when using the MyFood app [17].

### Implementation of the NUTREAT intervention

All participants, including patients, registered nurses, RDs, and leaders, found the NUTREAT intervention ***acceptable***. The patients found the number of recording days and periods satisfactory, and the MyFood app was considered user friendly. Registered nurses and RDs also expressed that they found the dietary recording notes in the EPR clear and understandable. Several systematic reviews have identified ease of use of digital health interventions as one of the most important facilitators to implementation [31,32].

The ***adoption*** was high among registered nurses, RDs, and leaders. Experienced advantage, compatibility with existing systems, and intervention complexity are considered important features of interventions that affect their adoption [33]. Additionally, both registered nurses and RDs reported that their leaders were positive and enthusiastic toward the intervention, arranging for intervention delivery to be seamless. Management support has previously been addressed as an important facilitator for HCPs to adopt new interventions [32].

Although adoption was high, few HCPs working with patients with HNC adhered to the intervention as it was prescribed, as only 1 registered nurse reported providing a referral to an RD for a patient with insufficient intake recorded in the MyFood app, despite a substantial proportion of patients not fulfilling ≥75% of their requirements for energy, indicating low ***fidelity****.* A possible reason for this could be high turnover among HCPs working with patients with HNC, which may lead to information about the study not being passed on due to loss of knowledge and experience [34]. For the future, this should be taken into consideration if conducting similar studies or implementing interventions into clinical practice to ensure that information is passed on to new staff, for example, through a mandatory online training and information program for new employees. Furthermore, the hospital at which the RCT was conducted underwent large organizational and structural changes during the study period, which may have affected the implementation of the NUTREAT intervention.

On the other hand, the NUTREAT intervention was considered ***appropriate*** by most of the participants. A key observation was that use of the MyFood app increased the patients’ awareness of their nutritional requirements. This also aligns with findings from the previous process evaluation study utilizing the MyFood system among hospitalized patients [17]. Furthermore, an Australian study investigating perceptions of patients using a technology-based intervention to participate in their own nutritional care found that patients experienced increased awareness of nutrition, being motivated to increase their dietary intake [35]. Patients found the intervention both relevant and valuable, although it was most useful during deterioration of eating ability. It is natural that the intervention would be perceived most valuable during these periods of dietary challenges, as the focus on nutritional intake is inherently heightened. In a commentary by Heneghan et al. [36], it was highlighted that in order to succeed making a significant impact on patient care, clinical trials should be designed and conducted in a manner that ensures that outcomes are relevant, appropriate, and of importance for patients in real-life settings.

Registered nurses and RDs found the NUTREAT intervention ***appropriate*** and useful in terms of preparing for patient follow-up prior to consultations. Perceived usefulness of digital health tools has previously been identified as an important facilitator for adoption among HCPs [32]. However, the interviews with the registered nurses and RDs identified that HCPs tended to point to others rather than themselves when asked who would be best suited to follow-up the dietary recordings from the MyFood app and that the leaders expressed concern about whether HCPs would prioritize reviewing the dietary recording notes. It has been highlighted that HCPs may experience that digital health interventions are time consuming and disrupt their existing workflow, which in turn may influence their adoption [32]. Another possible explanation is that the registered nurses consider monitoring of dietary intake as the RDs’ responsibility, whereas the RDs perceive that they already provide adequate nutritional follow-up for patients, making the intervention more useful for the registered nurses.

Patients, registered nurses, and RDs found the NUTREAT intervention ***feasible***, and most of the patients reported only needing initial training to be able to use MyFood. Furthermore, the participants experienced that the NUTREAT intervention was compatible with the treatment course for patients with HNC.

Furthermore, the present study revealed that the dietary recording notes did not always correspond to the patients’ self-reported intake. Possible explanations for this lack of correspondence between the MyFood recordings and reported intake could be recall bias when reporting intake to HCPs [37] or incomplete entries in the MyFood app. Moreover, previous studies that have evaluated MyFood recordings against validated reference methods have found that, although the agreement between the methods was high, the MyFood app underestimated the intake of certain food items [14,15]. Although some day-to-day variation is to be expected, this may affect the perceived credibility of the information obtained from the dietary recording notes. The perceived accuracy of data obtained from digital health technology may affect its adoption [32].

### The use of implementation strategies

Although the present study was not a strict implementation study, but rather utilized a hybrid type 1 design [18], it provided new insights regarding which implementation strategies were more relevant than others for implementing a complex intervention involving digital health monitoring.

A previous review on implementation strategies for introducing digital health interventions into patients’ homes indicated that *internal and external facilitation*, *audit and provide feedback*, *management* *support*, and *training of clinicians* are essential [38]. More recently, a systematic review investigating the use of implementation strategies for the integration of nutritional interventions in cancer care found that *audit and provide feedback* and *conduct educational meetings* were the most frequently used strategies [39]. In the present study, *audit and provide feedback* involved brief formal and informal meetings with the HCPs to discuss study progress and preliminary findings. This appeared useful for maintaining focus on the intervention and staying engaged with the intervention procedures. The strategy *remind clinicians* was operationalized by having a project worker available for the staff and offering access through a dedicated project phone, which helped clinicians quickly address questions regarding the intervention. *Conduct educational meetings* consisted of training sessions for registered nurses, physicians, and RDs, supporting a shared understanding of the intervention, and contributing to sustained attention among the HCPs. However, despite these efforts, ***fidelity*** did not increase.

Another implementation strategy included in the implementation plan was *recruit, designate, and train for leadership* [20]. The leaders played an important role in motivating and encouraging HCPs to follow the intervention; however, they did not take an active part in the intervention beyond this, which may have affected the fidelity among the HCPs. In a systematic review of reviews, Lau et al. [40] highlighted the importance of leadership in successful implementation. They emphasized the importance of identifying individuals who are trusted and respected by their staff to drive change from the start and noted that lack of leadership to advocate change and set priorities is an important barrier. Implementation success has also been shown to be greater in environments with supportive leaders who address individual concerns, encourage their employees, and are accessible, compared with organizations with limited leadership involvement [41]. Mechanisms by which leaders enhance implementation include being proactive, knowledgeable, supportive, and perseverant, as these characteristics foster a climate that facilitates adoption of evidence-based practices [42].

### Strengths and limitations

An important strength of this study is the inclusion of the perspectives of various respondents, including patients, registered nurses, RDs, and leaders involved in the follow-up of patients with HNC. Another strength is the application of established models and frameworks, such as the implementation outcomes described by Proctor et al. [28] and the ERIC implementation strategies [20]. The use of implementation frameworks is recommended both prior to and throughout implementation efforts and may help create a basis for developing knowledge about implementation that can be applied in various settings [43].

There are some limitations to our study. First and foremost, the number of registered nurses and leaders included in the interviews was limited. However, as we followed the “information power” principle [27], given a focused aim, sample specificity, and strong dialogue, a sample of 19 was considered sufficiently rich to provide meaningful insights into the implementation aspects explored. Second, physicians were not invited to participate due to insufficient information regarding which physicians had followed the intervention. The log of access to the dietary recording notes included only names, without indicating professional role, making it difficult to determine which physicians actually engaged with the intervention. Additionally, physicians see a large number of patients with HNC annually, and patient follow-up and staff rotation further complicated tracking their involvement. This lack of representation from the physician profession could have led to certain perspectives not being highlighted. For future studies, it would be important to plan ahead to identify which physicians are involved, for example, by recording professional role in the logs or surveying physicians about their participation. Another important limitation to our study is the available data regarding the outcome measure, *fidelity.* The intervention entailed both using and discussing information from the dietary recording notes in consultations with patients and providing additional referral to an RD if necessary. We were unable to investigate whether HCPs discussed the dietary recordings with patients during their consultations beyond the patients’ experiences. Furthermore, we were unable to capture the reason why HCPs did not provide an additional referral to an RD. However, a possible explanation may be that the patients were already in a treatment course where nutritional follow-up is a standardized part of the care. Finally, we acknowledge that the combination of data from the focus group and the individual interviews in the thematic analysis may limit the methodological distinction between data sources, although both addressed the same research questions and outcomes.

### Future implications

For the future, a trial rollout of the NUTREAT intervention should be carried out to avoid the perception among patients and HCPs that the intervention is only being introduced for a temporary period as part of a clinical trial. Moreover, the implementation of MyFood has been evaluated in various settings among both HCPs and patients of varying ages and diagnoses [16,44], showing high compliance among patients, indicating that the implementation among patients might be higher than among HCPs. In the future, specific consideration on how the intervention can be implemented among HCPs should be emphasized. Furthermore, roles should be clearly defined and responsibilities clearly delineated among HCPs to avoid displacement of responsibility across professional groups.

In conclusion, this study reported on a process evaluation to provide a greater understanding of the implementation of the NUTREAT intervention. Patients and HCPs found the intervention acceptable and appropriate, although fidelity was low among HCPs. The NUTREAT intervention increased awareness of nutritional requirements among patients and was considered helpful to HCPs in preparing for patient consultations. Given the fidelity challenges observed in this study, future research should pay specific consideration to identifying effective strategies for improving fidelity among HCPs. This would support both the quality of the delivery and the sustainability of the intervention in real-world settings.

## Author contributions

The authors’ responsibilities were as follows – FS, CV, LFA, CH, MMP: designed research; FS, MMP: conducted research; FS, CV, MMP: analyzed data; FS: wrote the paper; MMP: was the PI of the project and had the primary responsibility for the final content; and all authors read and approved the final manuscript.

## Data availability

Data described in the manuscript will be made available upon request pending.

## Declaration of generative AI and AI-assisted technologies in the writing process

During the preparation of this work, the authors used the University of Oslo's GPT-based language model service (GPT-4.1) in order to refine phrasing and improve clarity of English sentences. After using this tool, the authors reviewed and edited the content as needed and take full responsibility for the content of the publication.

## Funding

This study was funded by the Foundation Dam.

## Conflict of interest

LFA is a shareholder in FoodCapture AS which commercializes the MyFood system. All other authors report no conflicts of interest.
