# Supplementary material for: Implementation of Digital Seamless Nutrition Care throughout the Treatment Course for Patients with Head and Neck Cancer: A Process Evaluation
Source: Curr Dev Nutr. 2026 Mar 21;10(4):107677. doi: 10.1016/j.cdnut.2026.107677 (PMC13091737; doi:10.1016/j.cdnut.2026.107677)
Supplement: multimedia component 1 [file mmc1.docx]

Implementation plan – Nutrition Throughout the Treatment Course (NUTREAT)

# Part 1 – Assessment of current situation and planning the intervention

Phase 1 will be conducted prior to implementing the NUTREAT intervention. The project is a hybrid type 1 study. This includes:

*«… testing effects of a clinical intervention on relevant outcomes while observing and gathering information on implementation” (1). “In hybrid type 1 design, the primary focus is on testing the clinical intervention. The secondary focus is to explore implementation related factors” (2). “A hybrid type 1 design is indicated when the clinical effectiveness evidence remains limited, therefore studying implementation alone is premature” (2).*

To document that we follow the implementation strategies, we will keep a logbook and write minutes from meetings in the project group and with others involved in the project.

We will use implementation strategies from the Expert Recommendations for Implementing Change (ERIC) project (3), as presented in the tables below.

| **Strategy - Use evaluative and iterative strategies** | |
| --- | --- |
| Assess for barriers and facilitators | *“Assess various aspects of an organization to determine its degree of readiness to implement, barriers that may impede implementation, and strengths that can be used in the implementation effort” (3).*  Phase 1 will be conducted prior to implementing the NUTREAT intervention.   - Project team members (FS, CV, LFA, CH, MMP) will conduct a qualitative pre-implementation study including individual interviews with patients who have completed the treatment course for head and neck cancer, their family caregivers, and healthcare professionals working with patients with head and neck cancer.   - Preliminary findings from the interviews will be used to plan the intervention   - The study will be conducted from September 2023 to January 2024 and guided by the updated Consolidated Framework for Implementation Research (CFIR) - We will conduct a review of previously conducted studies using the MyFood tool to investigate already defined barriers and facilitators   - Leadership engagement – Leaders were not further involved in the process than informing registered nurses that they should use the MyFood app in previous study   - Lack of automatic integration of the MyFood system with the electronic patient record was an important barrier   - Use of champions was an important facilitator |

| **Strategy– Adapt and tailor to context** | |
| --- | --- |
| Tailor strategies | *“Tailor the implementation strategies to address barriers and leverage facilitators that were identified through earlier data collection” (3).*   - Review of previously conducted studies using the MyFood app   - Adapt strategies based on previous findings     - Alter the previously applied study design for studies involving the MyFood app:       - Previously dietary intake was only assessed in the intervention group, now we will also collect dietary intake data from the control group       - Intervention will be tailored to fit into the existing treatment course for HNC patients     - New method for transferring data from the dietary recordings in the app based on previous experiences that using the web-report is tedious for healthcare professionals       - Dietary intake data from the web-report will be copied into a dietary recording note in the electronic patient record     - To ensure that all patients are invited to participate regardless of their experiences with apps and their perceived fit to participate, registered nurses are instructed to invite all patients regardless of their preconditions (within the limits of the inclusion criteria) 🡪 this issue was also stressed in a previous evaluation study of the MyFood system     - To avoid that leaders are only involved in terms of informing registered nurses to use the MyFood app, we have planned for a meeting where also the leaders are given a demonstration of the MyFood app, and we will come to agreement on how the leaders’ engagement will be maintained.     - Continue the use of nurse champions as this has proven to be an important opportunity in previous study.     - We have scheduled a meeting with registered nurses in the hospital ward at December 6^th^ 2023 to provide information about the study |

# Part 2 – Preparations at the hospital and conducting educational meetings with healthcare professionals

Prior to rolling out the intervention preparations will be made at the hospital.

| **Strategy – Train and educate stakeholders** | |
| --- | --- |
| Conduct educational meetings | *“Hold meetings targeted toward different stakeholder groups (e.g., providers, administrators, other organizational stakeholders, and community, patient/consumer, and family stakeholders) to teach them about the clinical innovation” (3).*   - Meetings will be conducted with registered nurses at the outpatient radiation clinic (15/8 – 23, and 8/11 – 23)   - Provide information about the study and involve registered nurses in the planning   - Provide training in the MyFood app - Meetings will be conducted with registered nurses at the hospital ward (31/8- 23, and 6/12 – 23)   - Provide information about the study and involve registered nurses in the planning   - Provide training in the MyFood app - A meeting will be conducted with registered dietitians (7/11 – 23)   - Provide information about the study - Meetings will be conducted with physicians at the hospital (24/8 – 23 and 28/11 – 23)   - Provide information about the study and involve physicians in the planning   - Provide training in the MyFood app   *In a previous study about the MyFood system, empowerment among registered nurses was pointed out as an important strategy. Thus, it is important with thorough training in the MyFood app.* |
| Develop educational materials | *«Develop and format manuals, toolkits, and other supporting materials in ways that make it easier for stakeholders to learn about the innovation and for clinicians to learn how to deliver the clinical innovation” (3).*   - A procedure for participant recruitment has been developed to ensure a standardized recruitment process - Informational pamphlets about the study have been developed   - Information about the study and various study components, including an overview of when dietary recording periods will be conducted - An updated user manual for the MyFood app has been developed   - A step-by-step guide on how to navigate in the MyFood app |
| Distribute educational materials | *«Distribute educational materials (including guidelines, manuals, and toolkits) in person, by mail, and/or electronically” (3).*   - The procedure for participant recruitment will be distributed to registered nurses at the outpatient clinic by e-mail and printed out prior to intervention roll-out - Informational pamphlets about the study will be distributed by mail to registered nurses at the outpatient radiation clinic, registered dietitians, registered nurses at the hospital ward, and physicians - The user manual will be distributed by e-mail to registered nurses at the outpatient clinic and hospital ward, and physicians, in case they receive questions from patients regarding dietary recording |

| **Strategy – Develop stakeholder interrelationships** | |
| --- | --- |
| Identify and prepare champions | *“Identify and prepare individuals who dedicate themselves to supporting, marketing, and driving through an implementation, overcoming indifferences or resistance that the intervention may provoke in an organization” (3).*   - The registered nurses at the outpatient clinic will have the role as champions   - Only three registered nurses, working at various times, thus important to involve them all equally - 2-3 champions/registered nurses at the hospital ward   - A meeting is scheduled at the hospital ward on December 6^th^ – ask if anyone would like a more central role in the study   - Regular contact with these by mail and physically   - Champions will have the responsibility to follow-up that other registered nurses look into the recordings from the MyFood app in the patient record and use the information for patient follow-up, including providing additional referral to registered dietitian if necessary |
| Recruit, designate and train for leadership | *“Recruit, designate, and train leaders for the change effort” (3).*   - A meeting was held with the leader at the hospital ward to involve them in planning the study. Subsequently, a new meeting will be held to clarify roles - The head of the Head and neck oncology department will have the responsibility to pass on information about the study and assure that registered nurses are involved in the project |
| Use an implementation advisor | *“Seek guidance from experts in implementation” (3).*   - Cecilie Varsi will have the role as an implementation advisor for the research team and will contribute in the preparation phase, during the study, and with data analyses |

# Part 3 – Conducting the randomized controlled trial

For this part of the study, the randomized controlled trial will be rolled out.

| **Strategy – Use Evaluative and iterative strategies** | |
| --- | --- |
| Audit and provide feedback | *“Collect and summarize clinical performance data over a specified time period and give it to clinicians and administrators to monitor, evaluate, and modify provider behavior” (3).*   - Information regarding progression in recruitment and preliminary results will be given to involved healthcare professionals   - Information will be given in formal and informal meetings with involved healthcare professionals   - A newsletter will be sent regularly by e-mail to involved healthcare professionals throughout the study period |

| **Strategy – Provide clinical supervision** | |
| --- | --- |
| Provide local technical assistance | *“Develop and use a system to deliver technical assistance focused on implementation issues using local personnel” (3).*   - Frida Severinsen will act as a link between the healthcare professionals at the hospital, the developers of the MyFood app and the IT platform for secure data storage in case of technical issues throughout the study period |
| Conduct ongoing training | *“Plan for and conduct training in the clinical innovation in an ongoing way” (3).*   - Frida Severinsen will provide ongoing training in the MyFood app throughout the study period to registered nurses, physicians, and registered dietitians if necessary. |

| **Strategy – Support clinicians** | |
| --- | --- |
| Facilitate relay of clinical data to providers | *“Provide as close to real-time data as possible about key measures of process/outcomes using integrated modes/channels of communication in a way that promotes use of the targeted innovation” (3).*   - Information regarding how many patients are using the MyFood app at all times will be provided healthcare professionals throughout the study period   - Information will be sent by e-mail |
| Remind clinicians | *«Develop reminder systems designed to help clinicians to recall information and/or prompt them to use the clinical innovation” (3).*   - Frida Severinsen will regularly be physically present at the hospital and remind healthcare professionals of the study - Regular e-mail correspondence with leaders and involved healthcare professionals will be maintained throughout the study period to remind clinicians of the study |

**References**

1. Curran GM, Bauer M, Mittman B, Pyne JM, Stetler C. Effectiveness-implementation hybrid designs: combining elements of clinical effectiveness and implementation research to enhance public health impact. Med Care. 2012;50(3):217-26.

2. Landes SJ, McBain SA, Curran GM. An introduction to effectiveness-implementation hybrid designs. Psychiatry Res. 2019;280:112513.

3. Powell BJ, Waltz TJ, Chinman MJ, Damschroder LJ, Smith JL, Matthieu MM, et al. A refined compilation of implementation strategies: results from the Expert Recommendations for Implementing Change (ERIC) project. Implementation Science. 2015;10(1):21.
