# Supplementary material for: Implementation of Digital Seamless Nutrition Care throughout the Treatment Course for Patients with Head and Neck Cancer: A Process Evaluation
Source: Curr Dev Nutr. 2026 Mar 21;10(4):107677. doi: 10.1016/j.cdnut.2026.107677 (PMC13091737; doi:10.1016/j.cdnut.2026.107677)
Supplement: multimedia component 2 [file mmc2.docx]

Interview guide individual interviews

Individual interviews for the process evaluation of the NUTREAT intervention

Contents

[Registered nurses 2](#_Toc216779045)

[Leaders 7](#_Toc216779046)

[Patients 11](#_Toc216779047)

[References 14](#_Toc216779048)

# Registered nurses

| **Introduction** | *Information*: *Purpose of the interview – assess the experiences among registered nurses with the NUTREAT intervention (seamless nutrition care throughout the treatment course with main focus on the transitions between the outpatient clinic, home, and hospital).*   - Thank participant for their participation - Inform participant about the purpose of the interview   - Assess experiences with the NUTREAT intervention - Remind participant of the study procedure   - Patients with head and neck cancer are randomly allocated to receive the NUTREAT intervention or standard care   - The NUTREAT intervention involves monitoring of patients’ dietary intake from dietary record notes based on their self-recorded intake in the MyFood app, discussing the recorded intake, and providing additional referral to patients struggling to fulfil their estimated requirements - Emphasize that participant feedback is important for understanding their experiences with the intervention - Inform participant that the interview will be audio-recorded and that all data will be anonymized - Any questions before we begin? |
| --- | --- |
| **Opening question** | - Begin by asking about participant’s experiences with the intervention   - What has been challenging?   - How were any challenges solved?   - What was good about the intervention? - Ask the participant how the NUTREAT intervention was used in their daily work practice - Ask the participant how many patients they have been involved with in the intervention |
| **Acceptability**  *“The perception among stakeholders that a given treatment, service, or innovation is agreeable, palatable, or satisfactory. Acceptability should be assessed based on the stakeholder’s knowledge of or direct experience with various dimensions of the treatment to be implemented, such as its content, complexity, or comfort” (1)* | - Ask the particpant what results they have experienced from using the NUTREAT intervention   - Any results among the patients? How about healthcare professionals? - Ask the participant what has worked well and what has worked less well. If unclear or not answered, probe about:   - Ease of use/complexity   - Time and resource use   - Feasibility   - Workflow   - Role changes   - Benefits or drawbacks   - Technical issues   - Surprises - Ask the participant if patients have talked to them about the intervention   - If yes, ask how they perceived patients’ attitudes toward the NUTREAT intervention   - Ask if they have received any feedback from the patients, and if yes, ask what the feedback was - Ask the participant about how they perceive their manager’s attitudes toward the NUTREAT intervention   - Ask how they experienced the manager’s engagement and support, or lack thereof, being expressed     - Ask how this (either engagement or lack of engagement) has affected their own motivation and commitment to implementing the intervention   - Ask if the NUTREAT intervention has been discussed in meetings     - If yes, ask what has been said - Ask the participant about how they perceive their colleagues’ attitudes toward the NUTREAT intervention   - Ask if they have received any feedback from colleagues     - If yes, ask what the feedback was |
| **Adoption**  *“The intention, initial decision, or action to try or employ an innovation or evidence-based practice. May also be referred to as ‘uptake’” (1)* | *Information: When patients have completed dietary recording periods, designated dietary recording notes were created with information form the dietary recordings.*   - Ask if they have seen these dietary recording notes from dietary recording periods for the patients in the electronic patient record   - If yes, ask if they have accessed such dietary recording notes     - If no, ask why they have not accessed the dietary recording notes - Ask the participant if they have used the information from the dietary recording notes   - If yes, ask in what way and for what purpose they used the information   - If no, ask why the information has not been used   - Ask if they felt they knew how the information from the dietary recording notes was meant to be used     - If no, ask how this could have been made more clear     - If yes, ask if they referred patients with low recorded intake to a registered dietitian - Ask the participant about their opinion on the scope of the NUTREAT intervention   - Ask what they think of the number of recording days and recording periods for the patients |
| **Appropriateness**  *«The perceived fit, relevance, or compatibility of the innovation or EBP for a given practice setting, provider, or consumer; and/or perceived fit of the innovation to address a particular issue or problem”(1)*  **Fidelity**  *“The degree to which an intervention was implemented as it was prescribed in the original protocol or as it was intended by the program developers”(1)* | - Ask the participant about their experience of the format of the dietary recording notes in the electronic patient record   - Ask if there is anything that could have been done differently - Ask the participant whether they perceive the dietary recording notes to have any value   - If yes, ask in what way   - If no, ask in what way not - Ask the participant whether they perceive the NUTREAT intervention to have any value for the patients   *Information: Prior to rolling out the NUTREAT intervention, we conducted interviews with registered nurses, physicians, and patients to investigate their perceptions of the intervention (2). During these interviews, some concerns were raised regarding certain patient groups.*   - Ask the participant if they have experienced any patients having difficulties completing the intervention   - If yes, ask if there are any characteristics that these patients share - Ask the participant about whether they perceive the NUTREAT intervention to have any value for the follow-up they provide to patients   - Ask how they perceive the intervention affects the nutritional follow-up patients receive - Ask the participant how they think the NUTREAT intervention fits into the treatment course for patients with head and neck cancer now that they have seen patients follow the intervention for some time - Ask the participant to describe the intervention compared to standard follow-up   - Ask what is better or worse compared to standard follow-up - Ask the participant how they think the NUTREAT intervention fits in their daily work   - Ask how they perceived the workload of the NUTREAT intervention in their daily work - Ask the participants about their thoughts on resource and time use   - Ask how they perceive resources and time use in relation to the perceived benefit of the intervention - Ask if they had to make any changes to either the intervention or the way they work - Ask who they think the intervention is most suitable for, now that they have seen the intervention used with a range of patients - Ask the participant to describe their collaboration with the research team |
| **Conclusion** | *Information: Finally, we would like to know what can be learned from this study.*   - Ask the participant what could have been done differently to achieve better implementation of the NUTREAT intervention - Ask the participant what advice they would give if someone else were to implement the NUTREAT intervention |

# Leaders

| **Introduction** | *Information*: *Purpose of the interview – assess the experiences among leaders with the NUTREAT intervention (seamless nutrition care throughout the treatment course with main focus on the transitions between the outpatient clinic, home, and hospital)*   - Thank participant for their participation - Inform participant about the purpose of the interview   - Assess experiences with the NUTREAT intervention - Remind participant of the study procedure   - Patients with head and neck cancer are randomly allocated to receive the NUTREAT intervention or standard care   - The NUTREAT intervention involves monitoring of patients’ dietary intake from dietary record notes based on their self-recorded intake in the MyFood app, discussing the recorded intake, and providing additional referral to patients struggling to fulfil their estimated requirements - Emphasize that participant feedback is important for understanding their experiences with the intervention - Inform participant that the interview will be audio-recorded and that all data will be anonymized - Any questions before we begin? |
| --- | --- |
| **Opening question** | - Ask the participant to start by describing their experiences with the NUTREAT intervention   - Ask what they found challenging   - Ask how any challenges were addressed or resolved   - Ask what they found positive about the intervention |
| **Acceptability**  *“The perception among stakeholders that a given treatment, service, or innovation is agreeable, palatable, or satisfactory. Acceptability should be assessed based on the stakeholder’s knowledge of or direct experience with various dimensions of the treatment to be implemented, such as its content, complexity, or comfort” (1)* | - Ask the participant about the results they feel have been achieved by using the NUTREAT intervention   - If unclear, ask if they have seen any results in patients or healthcare professionals and to elaborate - Ask the participant what has worked well and what has worked less well. If unclear or not answered, probe about:   - Ease of use/complexity   - Time and resource use   - Feasibility   - Workflow   - Role changes   - Benefits or drawbacks   - Technical issues   - Surprises - Ask the participant about how healthcare professionals (specify relevant groups) have perceived the NUTREAT intervention while it has been ongoing   - Ask if they have received any specific feedback and what that feedback has been - Ask the participant if they have heard anything from patients regarding the NUTREAT intervention   - If yes, ask how they perceive patients’ attitudes toward the intervention   - Ask if they have received any feedback from patients and what that feedback has been |
| **Adoption**  *“The intention, initial decision, or action to try or employ an innovation or evidence-based practice. May also be referred to as ‘uptake’” (1)* | - Ask the participant about their thoughts on how the NUTREAT intervention could be implemented as a permanent part of the standard treatment course for patients with head and neck cancer - Ask the participant about how they exercise their leadership when new projects are to be implemented (e.g., do staff work independently, or do they need specific instructions?) - Ask the participant about how they perceive their role as leader affects the implementation of new solutions and interventions - Ask the participant if they have discussed the NUTREAT intervention with their staff in meetings   - If yes, ask what has been said   - Ask if any questions, comments, or feedback have come from staff, and if yes, ask them to elaborate |
| **Appropriateness**  *«The perceived fit, relevance, or compatibility of the innovation or EBP for a given practice setting, provider, or consumer; and/or perceived fit of the innovation to address a particular issue or problem (1)*  **Feasibility**  *“The extent to which a new treatment, or an innovation, can be successfully used or carried out within a given agency or setting. Typically invoked retrospectively as a potential explanation of an initiative’s success or failure, as reflected in poor recruitment, retention, or participation rates” (1)* | - Ask the participant how they think the NUTREAT intervention fits into the treatment course for patients with head and neck cancer now that they have seen patients and healthcare professionals follow the intervention for some time. - Ask the participant about the feasibility of their staff reading the dietary recording notes from the dietary recording periods   - Ask what they think about the scope of the NUTREAT intervention for healthcare professionals - Ask the participant how they perceive the intervention affects the nutritional follow-up that the patients receive - Ask the participant how they think the NUTREAT intervention fits into the daily work if healthcare professionals (e.g., registered nurses, physicians, registered dietitians)   - Ask what they think about the workload of the NUTREAT intervention in their daily work - Ask the participant about their thoughts on resources and time   - Ask how they perceive resources and time use in relation to the perceived benefit of the intervention - Ask whether they perceive the NUTREAT intervention to have value for the follow-up provided by healthcare professionals (specify relevant groups) to patients - Ask the participant to what extent they perceive healthcare professionals have followed up on the intervention   - Ask in what way - Ask whether they perceive the NUTREAT intervention to have value for patients   - Ask specifically about the number of recording days and periods in the MyFood app throughout the treatment course - Ask who they think the intervention is most suitable for now that they have seen the intervention used with a range of patients - Ask whether there are any healthcare professionals they think the intervention is more relevant for than others   - If yes, ask them to elaborate |
| **Conclusion** | *Information: Finally, we would like to know what can be learned from this study.*   - Ask the participant what could have been done differently to achieve better implementation of the NUTREAT intervention - Ask the participant what advice they would give if someone else were to implement the NUTREAT intervention |

# Patients

| **Introduction** | *Information*: *Purpose of the interview – assess the experiences among patients with the NUTREAT intervention (seamless nutrition care throughout the treatment course with main focus on the transitions between the outpatient clinic, home, and hospital)*   - Thank participant for their participation - Inform participant about the purpose of the interview   - Assess experiences with the NUTREAT intervention - Emphasize that participant feedback is important for understanding their experiences with the intervention - Inform participant that the interview will be audio-recorded and that all data will be anonymized - Any questions before we begin? |
| --- | --- |
| **Opening question** | - Ask the participant to start by describing their experiences with dietary intake throughout the treatment course, from time of diagnosis to present day   - Ask what challenges they may have encountered along the way   - Ask how challenges were addressed or resolved |
| **Experiences** | - Ask the participant about their experience of the follow-up they received regarding food and nutrition throughout the treatment course, including transitions between the hospital, outpatient clinic, and home   - If the participant mentions being hospitalized, ask about their experience during hospitalization and the transition from hospital to home   - Ask which different people were involved in supporting their dietary intake at the various locations (home, hospital, outpatient clinic)     - Prompt about healthcare professionals in the hospital, home care services, relatives, general practitioner, cancer coordinator, etc. - Ask the participant what they experienced as positive about the follow-up they received regarding food and nutrition throughout their treatment course   - Ask if there was anything they experienced as less positive and ask them to elaborate - Ask the participant how they perceived the focus on food and nutrition in the follow-up they received at the outpatient clinic and at home (or inpatient ward if hospitalized)   - Ask who they spoke to about food and nutrition throughout the treatment course     - Prompt about healthcare professionals, other patients, relatives, or no one   - If they spoke to no one, ask if this was something that they missed |
| **Participation in the study** | - Ask the participant to tell about their experiences participating in the study, receiving the NUTREAT intervention   - Ask about their experience of using the MyFood app as part of the follow-up on food and nutrition throughout the treatment course *(Acceptability)*     - Ask whether they found the MyFood app easy or difficult to use *(Acceptability)*   - Ask the participant in what way they felt the digital follow-up of food intake fit into their treatment course *(Appropriateness – compatibility)*      - Ask whether they perceived the digital follow-up as relevant for their treatment course and why or why not *(Appropriateness – relevance)*   - Ask the participant to describe how their motivation to participate in the intervention changed during the different phases of the treatment course   - Ask the participant how easy of difficult it was to remember using the MyFood app during the recording periods they were instructed to complete *(feasibility)* - Ask the participant about when during the treatment course they found digital nutritional follow-up to be most useful - Ask whether they perceived any value in using the MyFood app to record their dietary intake throughout their treatment course *(Appropriateness – usefulness)*   - If yes, in what way? - Ask if they learned anything from using the MyFood app   - If yes, ask what they learned - Ask if they received any help using the MyFood app   - If yes, ask who helped (e.g., healthcare professionals, relatives, home care services, cancer coordinator, general practitioner, etc.) - Ask their opinion on the number of recording days and periods in the MyFood app throughout the treatment course - Ask how they perceived the information they recorded in the MyFood app was followed up *(Fidelity)*   - Ask who followed it up (e.g., healthcare professionals, the patient themselves, relatives, etc.) - Ask if any actions were taken based on the recorded dietary intake in the MyFood app *(Fidelity)*   - If yes, ask who initiated these actions - Ask who they think this form of digital follow-up of dietary intake would be suitable for *(Appropriateness – perceived fit)* - Ask the participant whether they would recommend this kind of digital follow-up of dietary intake in the future   - If yes, ask to whom (e.g., healthcare services, other patients, relatives, etc.) - Ask the participant if they have any advice if the MyFood app was to be used by all patients in the future   - Ask if there is anything they think we should be careful about or pay attention to |
| **Summary** | - Ask the participant to summarize what they believe are the most important points discussed during the interview |

# References

1. Proctor E, Silmere H, Raghavan R, Hovmand P, Aarons G, Bunger A, et al. Outcomes for implementation research: conceptual distinctions, measurement challenges, and research agenda. Adm Policy Ment Health. 2011;38(2):65-76.

2. Severinsen F, Varsi C, Andersen LF, Henriksen C, Paulsen MM. Experiences with nutritional follow-up and barriers and opportunities of implementing digital seamless nutrition care in the head and neck cancer treatment course: a qualitative study from patient, family caregiver, and healthcare professional perspectives. BMC Health Serv Res. 2025;25(1):1358.
