# Supplementary material for: Implementation of Digital Seamless Nutrition Care throughout the Treatment Course for Patients with Head and Neck Cancer: A Process Evaluation
Source: Curr Dev Nutr. 2026 Mar 21;10(4):107677. doi: 10.1016/j.cdnut.2026.107677 (PMC13091737; doi:10.1016/j.cdnut.2026.107677)
Supplement: multimedia component 3 [file mmc3.docx]

Interview guide focus group discussion

Focus group discussion for the process evaluation of the NUTREAT intervention

| **Introduction** | *Information*: *Purpose of the interview – assess the experiences among registered dietitians with the NUTREAT intervention (seamless nutrition care throughout the treatment course with main focus on the transitions between the outpatient clinic, home, and hospital).*   - Thank participants for their participation - Inform participant about the purpose of the interview   - Assess experiences with the NUTREAT intervention - Remind participants of the study procedure   - Patients with head and neck cancer are randomly allocated to receive the NUTREAT intervention or standard care   - The NUTREAT intervention involves monitoring of patients’ dietary intake from dietary record notes based on their self-recorded intake in the MyFood app, discussing the recorded intake, and providing additional referral to patients struggling to fulfil their estimated requirements - Emphasize that participant feedback is important for understanding their experiences with the intervention - Inform participants that the interview will be audio-recorded, and that all data will be anonymized - Any questions before we begin? |
| --- | --- |
| **Opening question** | - Ask the participants to start by describing their experiences with the NUTREAT intervention   - Ask what they found challenging     - Ask how any challenges have been addressed or resolved   - Ask what they found positive about the intervention - Ask the participants about how they have used the NUTREAT intervention in their daily work - Ask the participants how many patients they have been involved with who were in the intervention group and received the NUTREAT intervention   - Ask them to provide an approximate number |
| **Acceptability**  *“The perception among stakeholders that a given treatment, service, or innovation is agreeable, palatable, or satisfactory. Acceptability should be assessed based on the stakeholder’s knowledge of or direct experience with various dimensions of the treatment to be implemented, such as its content, complexity, or comfort” (1)* | - Ask the participants about the results they feel have been achieved by using the NUTREAT intervention   - If unclear, ask if they have seen any results in patients or healthcare professionals and to elaborate   - Ask if they have experienced patients being referred to them as a result of low reported intake recorded in the MyFood app - Ask the participants what has worked well and what has worked less well   - If unclear or not answered, ask about ease of use, complexity, time and resource use, feasibility, workflow, role changes, benefits or drawbacks, technical issues, or surprises - Ask the participants if patients have talked to them about the NUTREAT intervention or the MyFood app   - If yes, ask how they perceive patients’ attitudes toward the intervention   - Ask if they have received any feedback from patients and what that feedback has been - Ask the participants how they perceive their leader’s attitude towards the NUTREAT intervention   - Ask how they experienced the leader’s engagement and support, or lack thereof, being expressed     - Ask how this (either expressed engagement or lack of engagement) has affected their own motivation and commitment to implementing the intervention   - Ask if the NUTREAT intervention has been discussed in meetings     - If yes, ask what has been said - Ask the participants how they perceive their physician/nurse colleagues’ attitudes toward the intervention   - Ask if they have received any feedback from other healthcare professionals and what that feedback has been |
| **Adoption**  *“The intention, initial decision, or action to try or employ an innovation or evidence-based practice. May also be referred to as ‘uptake’” (1)* | *Information: Upon completion of recording periods, designated dietary recording notes have been created in the electronic patient record.*   - Ask the participants if they have seen the dietary recording notes from dietary recording periods in the patients’ electronic patient records   - If yes, ask if they have accessed such dietary recording notes   - If no, ask why they have not accessed the dietary recording notes - Ask the participants if they have used the information from the dietary recording notes   - If yes, ask in which way and for what purpose they used the information   - If no, ask why the information was not used   - Ask if they felt they knew how to use the information from the dietary recording notes     - If no, ask how this could have been made more clear - Ask the participants about their opinion on the scope of the NUTREAT intervention   - Ask specifically what they think about the number of recording days and periods for the patients |
| **Appropriateness**  *«The perceived fit, relevance, or compatibility of the innovation or EBP for a given practice setting, provider, or consumer; and/or perceived fit of the innovation to address a particular issue or problem” (1)*  **Fidelity**  *“The degree to which an intervention was implemented as it was prescribed in the original protocol or as it was intended by the program developers”(1)* | - Ask the participants about their experiences with dietary recording notes in the electronic patient records   - Ask how they perceive the format of the dietary recording notes in the electronic patient record   - Ask if there is anything that could have been done differently - Ask the participants whether they perceive the dietary recording notes to have any value or benefit   - If yes, ask in what way   - If no, ask in what way not - Ask the participants whether they perceive the NUTREAT intervention to have any value for patients   *Information: Prior to rolling out the NUTREAT intervention, we conducted interviews with registered nurses, physicians, and patients to investigate their perceptions of the intervention (2). During these interviews, some concerns were raised regarding certain patient groups.*   - Ask the participant if they have experienced any patients having difficulties completing the intervention   - If yes, ask if there are any characteristics that these patients share - Ask the participants whether they perceive the NUTREAT intervention to have value for the follow-up they provide to patients   - Ask how they perceive that the intervention affects the nutritional follow-up of patients - Ask the participants how they think the NUTREAT intervention fits into the treatment course for patients with head and neck cancer now that they have seen patients follow the intervention for some time - Ask the participants how they would describe the NUTREAT intervention compared to standard care for patients with head and neck cancer   - Ask what is better or worse compared to standard follow-up - Ask the participants how they think the NUTREAT intervention fits into their daily work   - Ask how they perceive the workload of the NUTREAT intervention in their daily work - Ask the participants about their thoughts on resource and time use   - Ask how they perceive resource and time use in relation to the perceived benefits of the intervention - Ask if they have had to make any changes to either the intervention or the way they work - Ask the participants who they think the intervention is most suitable for, after seeing the intervention used with a range of patients - Ask how difficult they perceive the intervention to be to implement and how much training they needed - Ask the participants to describe their collaboration with the research team |
| **Conclusion** | - Ask the participants what they believe can be learned from this study   - Ask what could have been done differently to achieve better implementation of the NUTREAT intervention - Ask the participants what advice they would give if someone else were to implement the NUTREAT intervention |

1. Proctor E, Silmere H, Raghavan R, Hovmand P, Aarons G, Bunger A, et al. Outcomes for implementation research: conceptual distinctions, measurement challenges, and research agenda. Adm Policy Ment Health. 2011;38(2):65-76.

2. Severinsen F, Varsi C, Andersen LF, Henriksen C, Paulsen MM. Experiences with nutritional follow-up and barriers and opportunities of implementing digital seamless nutrition care in the head and neck cancer treatment course: a qualitative study from patient, family caregiver, and healthcare professional perspectives. BMC Health Serv Res. 2025;25(1):1358.
